# Shear wave elastography and Doppler ultrasound in kidney transplant recipients

**DOI:** 10.1590/0100-3984.2020.0148

**Published:** 2022

**Authors:** Luana Marinho Gonçalves, Gabriele Carra Forte, Tiago Garcia Holz, Lucas Lobraico Libermann, Carlos Eduardo Poli de Figueiredo, Bruno Hochhegger

**Affiliations:** 1 Pontifícia Universidade Católica do Rio Grande do Sul (PUCRS), Porto Alegre, RS, Brazil.

**Keywords:** Kidney transplantation, Elasticity imaging techniques, Ultrasonography, Doppler, color, Transplante renal, Técnicas de imagem de elasticidade, Doppler

## Abstract

**Objective:**

To evaluate the association between shear wave elastography parameters and arterial resistance in kidney transplant recipients.

**Materials and Methods:**

This was a prospective cross-sectional study involving consecutive adult kidney transplant recipients. All patients underwent color Doppler to evaluate the resistive index (RI) and ultrasound shear wave elastography for the quantification of renal allograft stiffness.

**Results:**

We evaluated 55 patients, of whom 9 (16.4%) had an RI defined as abnormal (≥ 0.79) and 46 (83.6%) had an RI defined as normal (< 0.79). The mean age was higher in the abnormal RI group than in the normal RI group (68.0 ± 8.6 years vs. 42.6 ± 14.1 years; *p* < 0.001), as was the mean shear wave velocity (2.6 ± 0.4 m/s vs. 2.2 ± 0.4 m/s; *p* = 0.013). Multivariate analysis identified two independent predictors of arterial resistance: age (OR = 1.169; 95% CI: 1.056 to 1.294; *p* = 0.003) and shear wave velocity (OR = 17.1; 95% CI: 1.137 to 257.83; *p* = 0.040).

**Conclusion:**

We observed an association between rigidity in the cortex of the transplanted kidney, as evaluated by shear wave elastography, and arterial resistance, as evaluated by color Doppler, in kidney transplant recipients.

## Abstract (pt)

**Objetivo:**

Avaliar a associação entre parâmetros de elastografia por onda de cisalhamento e resistência arterial em pacientes transplantados renais.

**Materiais e Métodos:**

Estudo transversal prospectivo. O estudo incluiu de forma consecutiva indivíduos adultos transplantados renais. Foram coletados dados demográficos e clínicos. Todos os pacientes foram submetidos à técnica ultrassonográfica para avaliação do índice de resistência (IR) e à quantificação da elasticidade do tecido por ondas de cisalhamento para avaliar a rigidez do aloenxerto renal.

**Resultados:**

Foram avaliados 55 pacientes. A média de idade e a velocidade da onda de cisalhamento foram maiores em pacientes com IR ≥ 0,79 (respectivamente, 68,0 ± 8,6 anos e 2,6 ± 0,4 m/s) quando comparados a pacientes com IR < 0,79 (respectivamente, 42,6 ± 14,1 anos, *p* < 0,001 e 2,2 ± 0,4 m/s, *p* = 0,013). A análise multivariada identificou a idade (OR = 1,169, IC 95%: 1,056 a 1,294; *p* = 0,003) e a velocidade da onda de cisalhamento (OR = 17,1, IC 95%: 1,137 a 257,83; *p* = 0,040) como fatores independentes associados a resistência arterial.

**Conclusão:**

Observou-se associação entre a rigidez do córtex do transplante renal avaliada por elastografia por onda de cisalhamento e a resistência arterial em pacientes pós-transplantados renais avaliados por Doppler.

## INTRODUCTION

Kidney transplantation is considered the procedure of choice for patients with end-stage renal disease, prolonging survival and reducing costs^(1)^. Worldwide, the incidence of end-stage renal disease in all age groups increased by 34.4% between 1990 and 2017^(2)^. In Brazil, there were more than 10 million newly reported cases of chronic kidney disease, as well as nearly 36,000 deaths related to the disease, in 2017. Due to its high cost, renal replacement therapy is still of limited availability in many regions of the world. In contrast, kidney transplantation is cost-effective, regardless of the donor type^(3)^.

Regarding the diagnosis of chronic nephropathy, renal biopsy remains the gold standard procedure. Nonetheless, there are biopsy-related risks, such as chronic allograft injury, and there is broad variation in interobserver agreement among pathologists^(4)^. In addition, in an outpatient setting, renal allograft function is assessed by using a multimodal approach, including Doppler ultrasound examination, clinical evaluation, and analysis of laboratory results. However, none of those tests alone is specific enough to detect incipient dysfunction of a renal allograft^(5)^.

Doppler ultrasound is currently the noninvasive method of choice for the evaluation of pathological alterations in the kidneys and has been widely used in the assessment of renal allograft status(^6^,^7^,^8^). Ultrasound has certain advantages, including the fact that it is readily available at most imaging centers, as well as having been proven safe and being a low-cost method(^6^,^7^). However, the usefulness of the resistive index (RI) in predicting acute rejection is still under debate because of its low diagnostic performance, which is due to the influence of extrarenal factors(^8^,^9^).

Morphological imaging of the renal allograft is performed mainly by B-mode ultrasound, computed tomography, or magnetic resonance imaging. However, structural evaluation of the renal parenchyma continues to be a challenge^(10)^. Ultrasound elastography has emerged as a tool to assist in the assessment of tissue stiffness, is already in use at major centers around the world, and has been the subject of a series of recent studies in the radiology literature of Brazil(^11^,^12^,^13^,^14^,^15^). Shear wave elastography (SWE) is the most effective noninvasive technique for assessing renal fibrosis after transplantation^(16)^ and assessing tissue velocity in response to a low-frequency pulse, a low velocity being indicative of tissue stiffness^(17)^. The use of SWE can reveal significant differences between allograft stability and acute or chronic allograft dysfunction^(16)^.

To our knowledge, there have been few studies evaluating the association between ultrasound elastography and Doppler ultrasound in kidney transplant recipients, especially in Brazil. Therefore, the aim of this study was to evaluate the association between SWE parameters and arterial resistance in kidney transplant recipients in Brazil.

## MATERIALS AND METHODS

This was a prospective cross-sectional study. The study protocol was approved by the Research Ethics Committee of the Pontifical Catholic University of Rio Grande do Sul (Reference no. 93226218.2.0000.5336), in the city of Porto Alegre, RS, Brazil. All of the authors signed a confidentiality agreement to ensure the anonymity of the data obtained from the electronic medical records of the hospital, and all participating patients gave written informed consent. The study was conducted in accordance with the guidelines set forth in the Declaration of Helsinki, and the article was prepared in accordance with The Strengthening the Reporting of Observational Studies in Epidemiology statement^(18)^.

The study included consecutive adult kidney transplant recipients who were referred to an outpatient nephrology clinic in southern Brazil for ultrasound imaging evaluation. Patients who had stage V chronic kidney disease and were on dialysis were excluded, as were those who had been diagnosed with hydronephrosis and those who were in their second or third trimester of pregnancy.

We collected demographic data (sex, age, and ethnicity) and clinical data (donor type, glomerular filtration rate, renal function, kidney size, and serum creatinine level). All patients underwent color Doppler to determine the RI and SWE for the assessment of renal allograft stiffness (quantification of the tissue elasticity). All ultrasound and SWE scans were performed by a board-certified radiologist, with eight years of experience, who was blinded to the clinical and biochemical profiles of the patients.

### Ultrasound examination

Ultrasound examinations were performed with a portable, stand-alone ultrasound system (Aplio 300; Toshiba Medical Systems, Otawara, Japan), a 3–5 MHz convex transducer, and a 5–14 MHz linear transducer. All patients were examined in the supine position. The length of the transplanted kidney was measured by B-mode ultrasound. Color Doppler was used in order to evaluate tissue perfusion and to calculate the RI for the transplanted kidney:

$$\mathrm{RI}=\left(\mathrm{peak}\;\mathrm{systolc}\;\mathrm{velocity}-\mathrm{end}-\mathrm{diastolic}\;\mathrm{velocity}\right)/\mathrm{peak}\;\mathrm{systolic}\;\mathrm{velocity}$$

**Figure 1:**
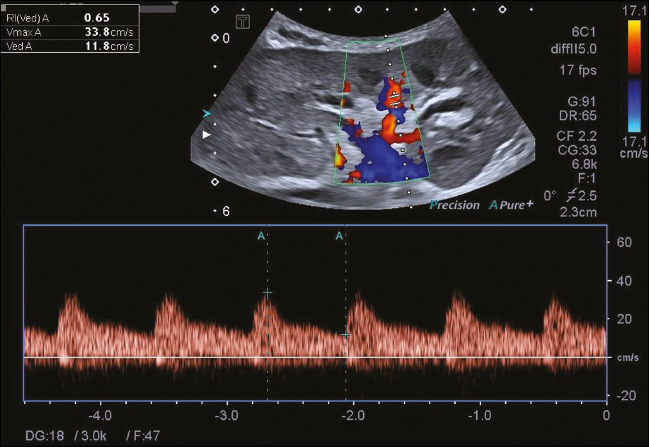
Spectral color Doppler for evaluation of the RI of the interlobar artery.

Intrarenal Doppler of the segmental arteries was performed at three representative locations (Figure 1), and the average was used for statistical analysis.

### Kidney stiffness measurement

**Figure 2:**
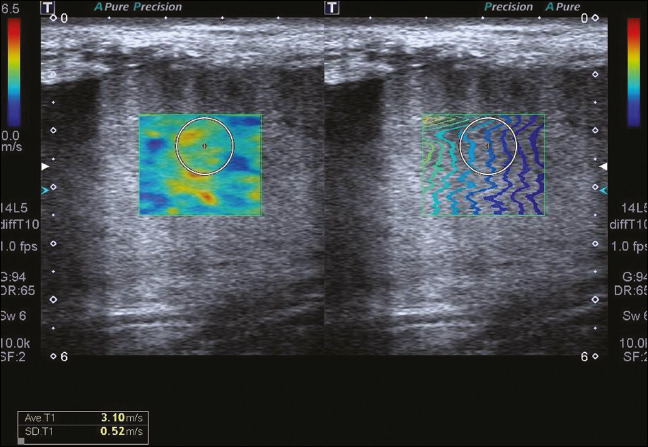
SWE of the right parenchyma of a renal allograft.

The SWE method generates shear waves within the tissue by using the radiation force of a focused ultrasound beam, and the propagation of the shear wave is monitored in order to quantify tissue stiffness. Young’s modulus, measured in kilopascals, is the measure used for allograft stiffness, in which higher values correlate with a higher degree of fibrosis(^19^,^20^,^21^). The SWE measurements were obtained in a single region of interest (ROI): an area of renal parenchyma. The distance from the skin to the ROI was recorded as kidney depth. All patients were examined between 8:00 a.m. and 10:00 a.m., on an empty stomach and in the supine position. The study was conducted under controlled conditions. The ROI was positioned perpendicular to the surface closest to the skin, with minimal compression of the transducer to avoid external compression of the transplanted kidney, followed by activation of the tissue image quantification mode with the acquisition of the shear wave velocity in the ROI, which was displayed on a color-coded map. Thirteen quantification cursors were placed within the ROI in the renal cortex, each providing the values for shear wave velocity (in meters per second) and elasticity (in kilopascals). The stiffness of the renal cortex of the transplanted kidney was calculated by determining the average values for the shear wave velocity and elasticity (Figure 2).

### Sample size

Using the effect size estimated in the study conducted by Chiocchini et al.^(22)^, a total width of 0.15, a two-sided type I error of 0.05, and a power of 0.80, we calculated the sample size required to be 55 subjects (G * Power software version 3.1.9.2).

### Statistical analysis

The statistical analysis was performed with the Predictive Analytics Software package, version 18.0 (SPSS Inc., Chicago, IL, USA). Quantitative variables are expressed as mean ± standard deviation or as median (interquartile range [IQR]), whereas qualitative variables are expressed as absolute and relative frequencies. For analysis purposes, patients were categorized into two groups by renal RI: < 0.79 (normal, n = 46) and ≥ 0.79 (abnormal, n = 9). A multivariate analysis was performed by using binary logistic regression with generalized linear models to identify the independent variables associated with the Doppler RI. The level of significance was set at *p* < 0.05.

## RESULTS

Between September 2018 and July 2019, 55 patients were enrolled in the study. The mean age was 46,0 ± 16.4 years; 38 (69.1%) of the patients were male, and 44 (80%) self-identified as White. Table 1 summarizes the characteristics of the study sample. The median values for glomerular filtration rate and creatinine were 17.3 mL/min (IQR, 8.4–28.4 mL/min) and 4 mg/day (IQR, 2.3–6.1 mg/day), respectively. Forty-one patients (74.5%) had advanced chronic kidney disease or end-stage renal disease, and 53 (96.4%) had impaired renal function. Of the 55 patients evaluated, 52 (94.5%) received a kidney from a deceased donor. The mean RI of the main renal artery and mean shear wave velocity were 0.65 ± 0.08 and 2.3 ± 0.4 m/s, respectively.

**Tabela:** General characteristics of the kidney transplant recipients evaluated.

Variable	(N = 55)
Age (years), mean ± SD	46.0 ± 16.4
Male sex, n (%)	38 (69.1)
Race, n (%)	
White	44 (80.0)
Black	11 (20.0)
Donor, n (%)	
Deceased	52 (94.5)
Living	3 (5.5)
eGFR (mL/min/1.73 m^2^), median (IQR)	17.3 (8.4–28.4)
Creatinine (µmol/L), median (IQR)	4.0 (2.3–6.1)
Renal function impairment, n (%)	
Mild/moderate	14 (25.5)
Severe	18 (32.7)
End-stage	23 (41.8)
Transplanted kidney size (cm), mean ± SD	11.4 ± 1.2

SD, standard deviation; eGFR, estimated glomerular filtration rate.

Table 2 shows a comparison between the normal RI and abnormal RI groups in terms of the patient characteristics. The mean age was higher in the abnormal RI group than in the normal RI group (68.0 ± 8.6 years vs. 42.6 ± 14.1 years, *p* < 0.001), as was the mean shear wave velocity (2.6 ± 0.4 m/s vs. 2.2 ± 0.4 m/s, *p* = 0.013). There were no significant differences between the two groups regarding sex, race, estimated glomerular filtration rate, serum creatinine, graft size, and kidney donor type. Multivariate analysis identified two independent predictors of arterial resistance: age (OR = 1.169; 95% CI: 1.056 to 1.294; *p* = 0.003) and shear wave velocity (OR = 17.1; 95% CI: 1.137 to 257.83; *p* = 0.040).

**Table:** Comparison between the kidney transplant recipients with normal and abnormal RIs (< 0.79 and = 0.79, respectively), in terms of sociodemographic and clinical variables.

Variable	RI < 0.79 (n = 46)	RI = 0.79 (n = 9)	*P*
Age (years), mean ± SD	42.6 ± 14.4	68,0 ± 8.7	< 0.001
Creatinine (µmol/L), median (IQR)	4.0 (2.3–6.5)	3.8 (2.3–5.8)	0.935
eGFR (mL/min/1.73 m^2^), median (IQR)	17.3 (7.9–30.8)	16.5 (9.2–25.4)	0.806
Transplanted kidney size (cm), mean ± SD	11.5 ± 1.2	11.1 ± 0.8	0.305
Shear wave velocity (m/s), mean ± SD	2.2 ± 0.4	2.6 ± 0.4	0.038

SD, standard deviation; eGFR, estimated glomerular filtration rate.

## DISCUSSION

We identified a positive association between SWE parameters and arterial resistance in kidney transplant recipients. In addition, age and shear wave velocity were found to be independent predictors of arterial resistance. These findings indicate that SWE can not only quantify tissue elasticity in a renal allograft but also predict arterial resistance.

Determination of the RI is a useful tool in assessing the prognosis for graft survival in the chronic period after transplantation, because it reflects the vascular status of the allograft(^23^,^24^,^25^,^26^). An RI > 0.8 are usually typical of allograft malfunction and death with a functioning graft^(27)^. Ba et al.^(24)^ found that an increase > 1 in the RI could reflect absence of end-diastolic blood flow, resulting in a worse prognosis for renal allograft survival. Recent studies of the extrarenal factors influencing the RI in kidney transplant recipients (e.g., recipient age, arterial stiffness, and left ventricular diastolic function) have demonstrated that the RI is dependent on the vascular characteristics of the recipient(^28^,^29^).

Although color Doppler is an established method to assess renal allografts, ultrasound elastography seems to show renal impairment earlier. Kidney stiffness has been used as a predictor of renal allograft fibrosis(^30^,^31^,^32^,^33^,^34^). Renal sampling by SWE has been shown to have a reliability of 97.6%^(22)^. In a study conducted in Turkey, renal parenchymal stiffness correlated positively with the RI^(33)^. In a study of 50 kidney transplant recipients under clinical suspicion of allograft fibrosis in Italy, a strong correlation was observed between low tissue elasticity and moderate-to-severe interstitial fibrosis^(35)^. In addition, several studies have indicated that an increase in shear wave velocity is predictive of a worsened estimated glomerular filtration rate, as well as renal fibrosis, in kidney transplant recipients(^36^,^37^,^38^).

Our study has some limitations. First, the post-transplant period was not evaluated. In addition, no biopsies were performed, so we were unable to compare our results with the biopsy findings. Furthermore, all of the results were interpreted by the same examiner. Moreover, there was no control group of healthy patients to rule out the possibility that the advanced age of the patients in our sample was the cause of the abnormal stiffness of the renal parenchyma. However, to our knowledge, this is the first study to investigate the association between renal stiffness, as assessed by SWE, and arterial RI, as assessed by ultrasound Doppler, in kidney transplant recipients in Brazil.

In conclusion, the stiffness of the renal cortex of a transplanted kidney appears to correlate with arterial resistance in kidney transplant recipients. In addition, monitoring renal allografts by SWE may provide an additional early prognostic index to assess chronic renal dysfunction.
